# Improvement of epidermal covering on AEC patients with severe skin erosions by PRIMA-1^MET^/APR-246

**DOI:** 10.1038/s41419-020-2223-8

**Published:** 2020-01-16

**Authors:** Edith Aberdam, Lauriane N. Roux, Philippe-Henri Secrétan, Franck Boralevi, Joël Schlatter, Fanny Morice-Picard, Stefano Sol, Christine Bodemer, Caterina Missero, Salvatore Cisternino, Daniel Aberdam, Smail Hadj-Rabia

**Affiliations:** 1 INSERM U976, Paris, France; 2 Université Paris-Diderot, Paris, France; 3 Department of Pharmacy, Hôpital Universitaire Necker-Enfants Malades, Paris, F-75006 France; 4 Faculté de Pharmacie, EA 401, Université Paris Sud, Chatenay-Malabry, F-92290 France; 5 Pediatric Dermatology unit, National Centre for Rare Skin Disorders, Hôpital Pellegrin-Enfants, CHU de Bordeaux, Bordeaux, F-33000 France; 6 CEINGE, Napoli, Italy; 7 University of Naples Federico II, Napoli, Italy; 8 Department of Dermatology and Reference center for Genodermatoses and Rare Skin Diseases (MAGEC), Paris, F-75006 France; 9 Institut Imagine, Hôpital Universitaire Necker-Enfants Malades, APHP5, Paris, F-75006 France; 10 Université Paris Centre, INSERM U1144, Paris, France; 11 Faculté de Pharmacie de Paris, INSERM U1144, Paris, France

**Keywords:** Mechanisms of disease, Translational research

## Abstract

P63 is a major transcription factor regulating skin development and homeostasis. It controls many genes involved in cell proliferation, adhesion, and early differentiation. P63 is mutated in several rare syndromes called p63-related ectodermal dysplasia syndromes (ED). The main forms are EEC and AEC syndromes due to p63 missense mutations on the DBD and SAM domains, respectively. ED patients display many developmental defects, including ectrodactyly, clef/lip palate, and ectodermal dysplasia, while AEC patients suffer from severe skin erosions that not always heal. We have previously showed that ED-derived iPSC display altered epidermal commitment. P63 belongs to the p53 gene family sharing similar structural domains. We found that ED-iPSC epidermal commitment can be rescued by a p53-reactivating compounds called PRIMA-1^MET^, also named APR-246 and currently used in anticancer clinical trials. Here, we established primary epidermal culture from two AEC children (S.F. and Y.M.) suffering from persistent skin erosions at age of 9 and 15, respectively. These patients carry missense mutations on the SAM domain (I576T and I537T). We found that primary keratinocytes (KCs) isolated from these AEC patients underwent altered epidermal differentiation that was rescued by PRIMA-1^MET^ treatment. It prompted us to formulate the compound onto a cream that was topically applied on the right hand of one patient and on the scalp of the second patient. In both cases, the daily treatment allowed re-epithelialization of the eroded skin and a drastic loss of pain after few weeks, improving quality of life. Normally, mutant p63 exerts a dominant-negative effect, mainly through the formation of aggregate with WT p63 and p73. PRIMA-1^MET^ did not reduce protein aggregation while enhancing cell differentiation, suggesting that PRIMA-1^MET^ targets cell differentiation and not p63 activity directly. In conclusion, we propose that repurposing of the antitumoral PRIMA-1^MET^ compound could become a general treatment of AEC skin erosions.

## Introduction

*TP63*, member of the P53 gene family, encodes for P63 protein, a master regulator of embryonic steps of epithelial development. P63 is essential for epithelial homeostasis, mainly controlling the proliferative potential of epidermal stem cells and the stratification of epithelial structures^1–3^. Deletion of p63 in mice results in the complete absence of stratified epithelia in organs, such as the epidermis, breast, prostate, and bladder^4–6^. Heterozygous missense mutations in *TP63* gene are associated to P63-related ectodermal dysplasia (ED). Two overlapping phenotypes are more frequent: 1/ankyloblepharon-ectodermal dysplasia-clefting syndrome (AEC, MIM 106260), characterized by ectodermal dysplasia, including alopecia, scalp erosions, dystrophic nails, hypodontia, ankyloblepharon, and cleft lip and/or cleft palate; 2/ectrodactyly-ectodermal dysplasia-cleft lip/palate (EEC, MIM 604292), which differs from AEC by the ectrodactyly and absence of scalp erosions. A certain degree of genotype-to-phenotype correlation is reported. EEC mutations are clustered in the DNA-binding domain, while AEC mutations are found in the sterile α-motif or transactivation-inhibitory domain^7^. Consistent with the dominant inheritance pattern, there is an agreement that these mutations have a dominant-negative effect by interfering with p63 DNA binding^8^.

While there is no curative treatment, care and follow-up of AEC and EEC patients are challenging and require multidisciplinary and expert, surgical, and medical teams for the clefting surgery, hearing aid, and ocular long-term follow-up. For that purposes, skin integrity is mandatory. For example, scalp erosions frequently interfere and delayed surgery. While spontaneous skin healing occurs within the first 2 years of life, in a number of patients, skin erosions extended to the back, the palms and soles and persist beyond 2 years. By the use of EEC/AEC-derived induced pluripotent stem cells (iPSC), we have previously shown that ED-iPSC displayed altered epidermal commitment that can be rescued by a P53-reactivating compounds called PRIMA-1^MET^, also named APR-246 and successfully tested in antitumor clinical trials^9,10^ (https://www.aprea.com/pipeline/apr-246/). Here, we show the significative improvement of chronic skin erosions in two AEC patients by topical use of PRIMA-1^MET^.

## Results and discussion

### Phenotype and genotype description of AEC patients

**Fig. 1:**
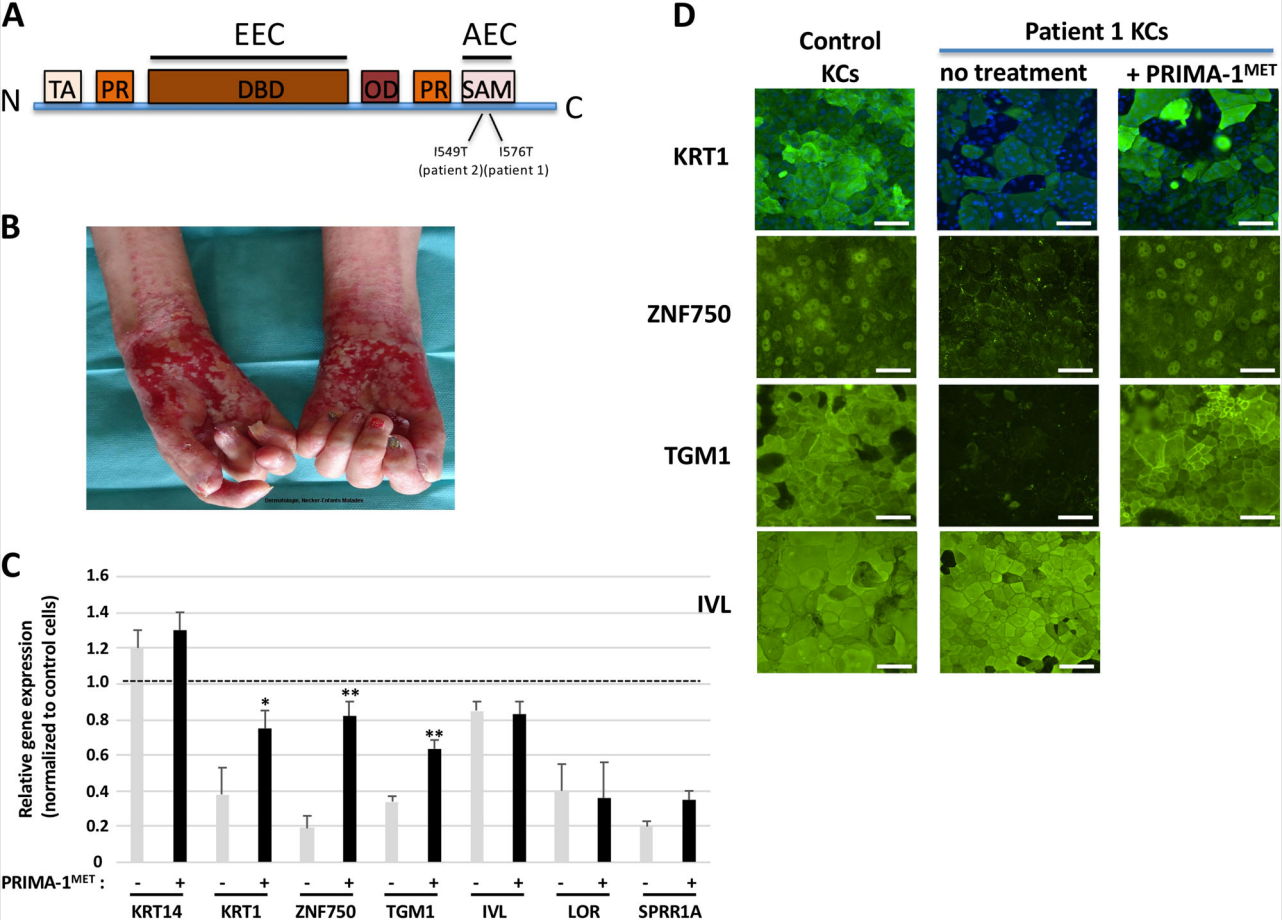
Altered epidermal differentiation of patient 1 KCs rescued by PRIMA-1^MET^. Structure of *TP63* gene and location of the identified mutations on the SAM domain for patients 1 and 2 (**a**). Patient 1 displays hand skin erosions (**b**). Patient 1 KCs are not differentiating properly, but can be rescued by PRIMA-1^MET^ treatment, as illustrated by qRT-PCR analyses (*n* = 3). One-way ANOVA followed by Dunnett’s test was performed *p < 0.05, **p < 0.01, (**c**) and immunofluorescence stainings (*n* = 4) (**d**) of specific markers of differentiation. Scale bar: 100 μm. DBD DNA-binding domain, IVL involucrin, KC keratinocytes, KRT keratin, OD oligomerization domain, PR proline domain, SAM sterile alpha domain, TA transactivation domain, TGM1 transglutaminase 1, ZNF750 zinc finger protein 750, ILV involucrin, LOR loricrin, SPRR1A small proline-rich protein 1A (or cornifin-A).

Patient 1 is a 9-year-old daughter of healthy non-consanguineous parents. She was referred at birth for cleft lip and palate and scalp erosions. Pregnancy and delivery were uneventful. AEC syndrome was confirmed by the identification of a missense mutation in the SAM domain of *TP63* gene (c.1727T > C; p.Ile576Thr) (Fig. 1a). Few months after birth, she developed bilateral and painful erosions of the palms and soles that never completely healed. For 9 years, several therapeutic attempts were proposed, based on specific dressings and topical preparations and opiate analgesics. Skin grafting was discussed. Painful retraction of the fingers and sole involvement impact fine motor skills (writing, drawing, clothing…) and walking, respectively.

Patient 2 is a 15-year-old female patient born to healthy non-consanguineous parents. AEC syndrome was confirmed by the identification of a missense mutation in the SAM domain of *TP63* gene c.1610T > C, p.Ile537Thr. She presented at birth with cleft of the palate and ankyloblepharon. She had abnormal nail and teeth, and superficial recurrent erosions of the fingers. Since birth, she presented with complete aplasia cutis of the scalp responsible for chronic anemia. Despite all medical efforts, scalp aplasia never healed and gradually spread to the forehead and ears. She was treated by daily dressings. She received paracetamol before and after the dressing. Pain comparison with visual analog scale was measured at 6 during the dressings.

### Establishment of primary AEC epidermal cell lines and epidermal differentiation

Biopsy was undertaken from on nonerosive region of the skin in patient 1. Primary epidermal culture was established and amplified. No difference was observed between WT and AEC KCs proliferation or cell death (not shown). Stratification/differentiation was induced by raising calcium concentration of the medium to 1.5 mM. Cells were maintained for 10 days, and both gene and protein expression for specific differentiated epidermal markers were performed, respectively, by qRT-PCR and immunostaining analysis. Expression of cytokeratin 1 (KRT1), ZNF750, transglutaminase (TGM1), loricrin (LOR) and small proline-rich protein 1A (SPRRA1) genes were reduced in AEC keratinocytes (KCs) of patient 1 as compared with wild-type (WT) KCs (Fig. 1c). It suggests a delayed or altered epidermal differentiation in patient cells. Except for cornified markers (LOR and SPRRA1), the expression of these genes was restored by PRIMA-1^MET^. Accordingly, patient 1 differentiated KCs displayed by immunofluorescence staining reduced nuclear ZNF750, pericellular transglutaminase, and cytoplasmic KRT1, as compared with normal KCs (Fig. 1d). Remarkably, in the presence of PRIMA-1^MET^, these markers were efficiently rescued in AEC KCs at day 10 (Fig. 1d). This strongly suggests that PRIMA-1^MET^ efficiently rescued AEC cell differentiation. Of interest, while KRT1, transglutaminase, and ZNF750 were profoundly altered in AEC-differentiated KCs, involucrin (IVL) was normally expressed (Fig. 1c, d). It has been shown that involucrin does not behave like the other skin markers in wound and in psoriasis^11,12^. It would be interesting to study this difference in the context of AEC.

### p63 aggregation is not reduced by PRIMA-1^MET^

**Fig. 2:**
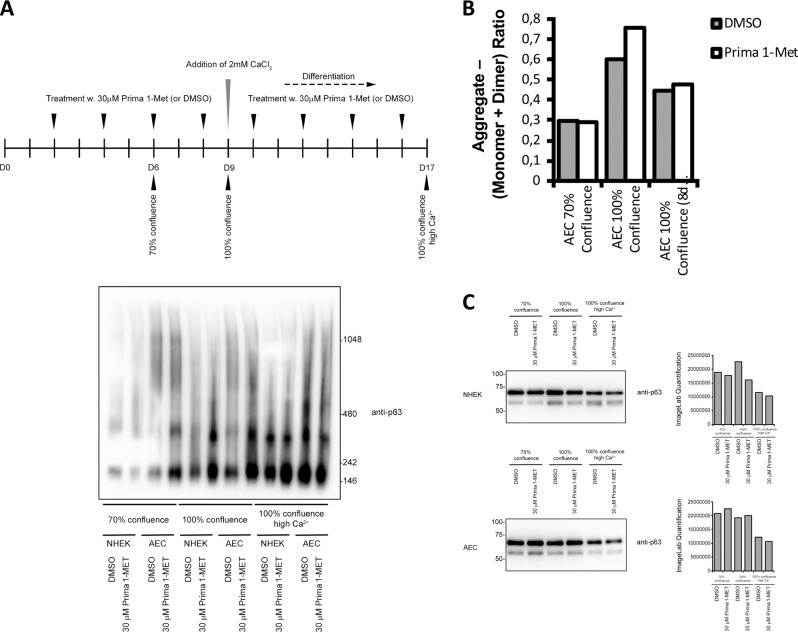
Protein aggregation is induced in differentiated patient 1 KCs, but not reduced by PRIMA-1^MET^. **a** Protein extracts were collected from WT and patient 1 KCs (AEC) at confluence and after 10 days of differentiation and loaded onto non-denaturing gel for western blot analysis with p63-specific antibody (**b**). Representative of three independent experiments. Quantification of the bands (**c**). Equivalent amount of protein was loaded for each condition.

Mutant AEC p63 exerts a dominant-negative effect, mainly through the formation of aggregates with WT p63 and also with the p53 family member p73^13^. We tested whether PRIMA-1^MET^ rescue could be due to reduction of protein aggregation. Untreated and treated mutant and WT epidermal cells were lysed in native conditions for total protein extraction. Protein extracts were then loaded on a non-denaturing gel followed by transfer for western blot analysis with an anti-p63-specific antibody. Protein aggregation was detected in AEC-differentiated cells and not in WT cells, as expected. However, while PRIMA-1^MET^ was able to rescue epidermal differentiation and corresponding specific gene expression, it did not abolish protein aggregation driven by the mutant p63 molecule (Fig. 2). This is in contrast with its disaggregation activity on mutant p53^14^. It strongly suggests that PRIMA-1^MET^ targets epidermal cell differentiation, but not p63 activity. This could suggest a more general effect of this small molecule on defective wound healing, like in keloids, leg ulcers, or diabetic ulcers.

### Improvement of epidermal covering

**Fig. 3:**
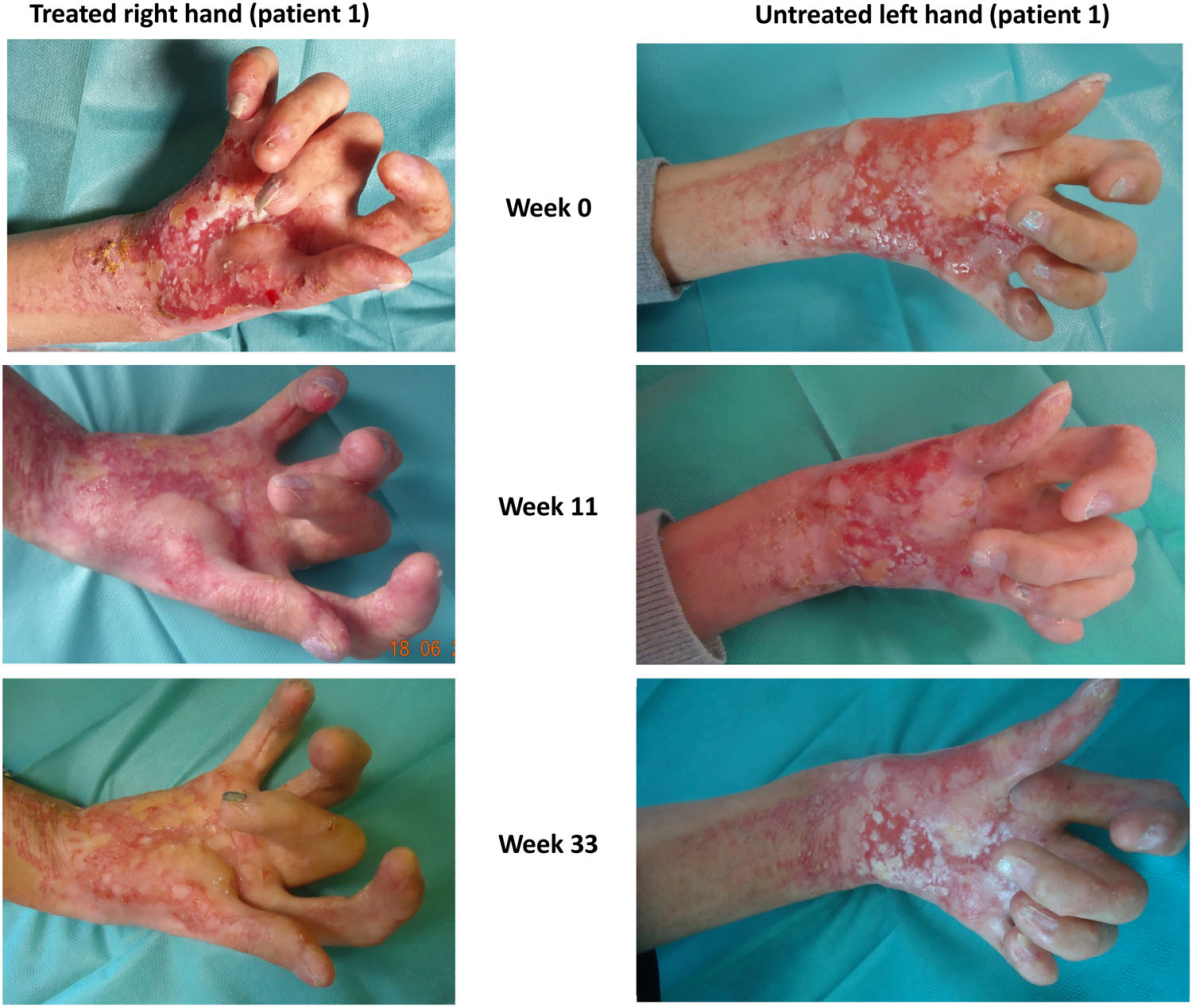
Topical treatment of the patient 1 erosions with formulated PRIMA-1^MET^. Formulated PRIMA-1^MET^ was applied daily on the right palm of the patient 1 (left), while the left palm was treated with the vehicle alone as a control (right).

On the basis of the in vitro results and the ongoing phase II PRIMA-1^MET^/APR-246 trials, we were authorized by the French Agency for Health and Drugs (ANSM) to administrate PRIMA-1^MET^ to two patients under the responsibility of the dermatologist and the pharmacist. PRIMA-1^MET^ was formulated onto a cream (see the Materials and methods section for compound formulation), and first applied topically on the skin of Balb/C mice for 1 week without any sign of irritation or cytotoxicity (not shown). After informed consent, patient 1 received 5 mg daily of PRIMA-1^MET^ in SERAQUA vehicle on skin erosions. Right palm was treated daily, while the left palm was treated with the vehicle alone. After 11 weeks, the daily treatment allowed re-epithelialization of the skin erosions and a drastic improvement of pain, leading to stop painkillers (Fig. 3). At week 33, epidermalization was almost complete, but not fully cornified (Fig. 3).

**Fig. 4:**
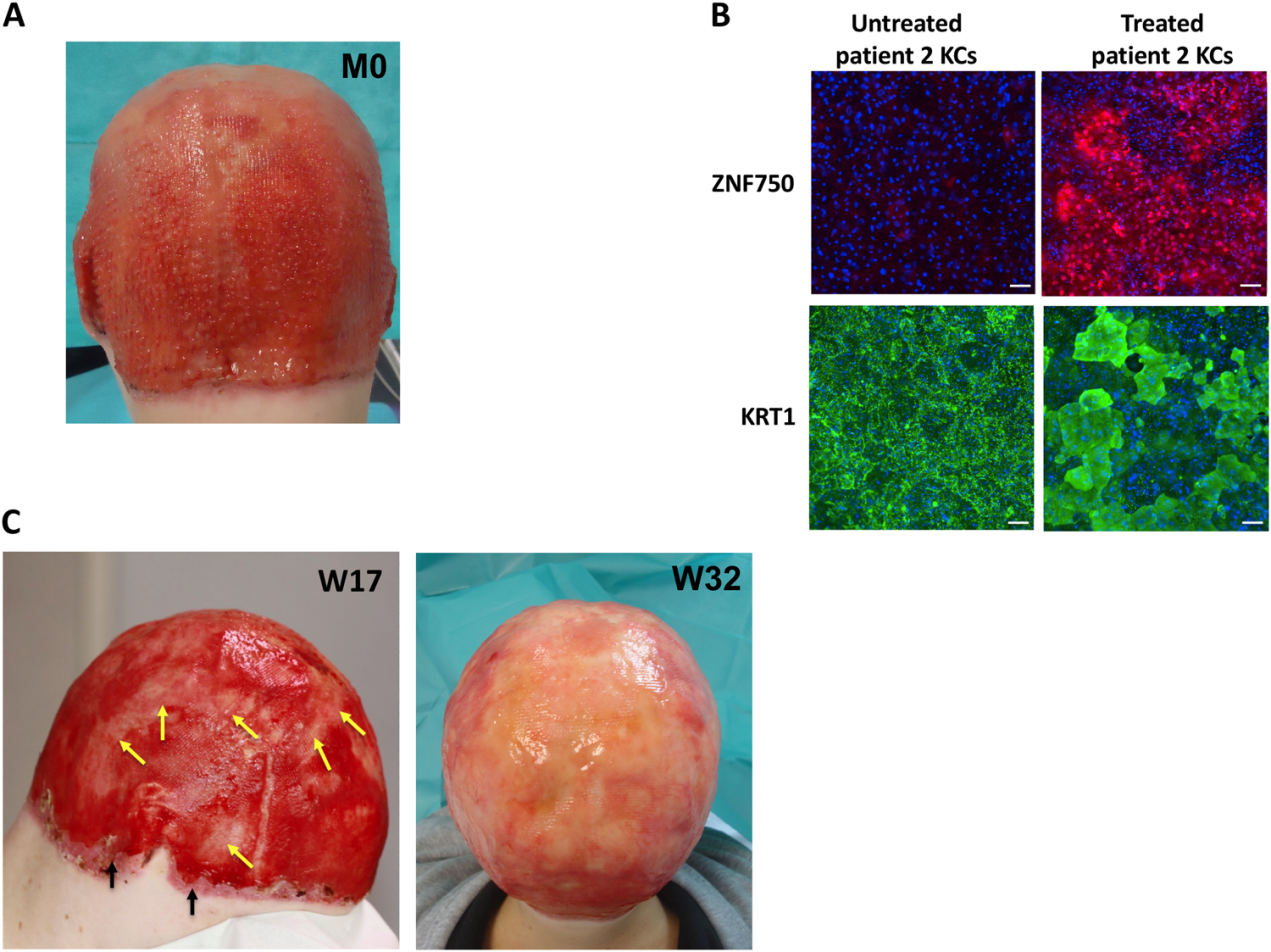
Patient 2 presented with severe persistent scalp erosion and altered KCs differentiation reverted by PRIMA-1^MET^ treatment. **a** Patient 2 displays skin erosion of the scalp. **b** Altered patient 2 KCs differentiation was rescued by PRIMA-1^MET^ as illustrated by immunostaining analyses; scale bar: 100 μm. **c** Formulated PRIMA-1^MET^ was applied daily on the whole surface of the scalp of patient 2. First improvement was seen during the first 5 weeks of treatment. First epidermalization areas were seen at week 4 (not shown). Confluence of such areas was seen at week 17 (yellow arrows). Remarkably, the whole peripheric scalp was completely re-epidermized (back arrows). Most of the scalp became covered by week 32. KC keratinocytes, KRT keratin, ZNF750 zinc finger protein 750.

Based on these achievements, a biopsy was undertaken from a on nonerosive region of the skin in patient 2, suffering from an entire scalp erosion (Fig. 4a). As observed with KCs from patient 1, KCs from patient 2 displayed similar epidermal stratification/differentiation alteration that was rescued by PRIMA-1^MET^ (Fig. 4b). Then, patient 2 was treated on the entire scalp erosion. Improvement of the scalp erosion was already observed after 5 weeks of topical treatment (Fig. 4c). Within the first 3 weeks, oozing decreased. Epidermal growth was seen with normal skin in some areas that progressively merge. At week 32, the scalp was largely recovered (Fig. 4c), and dressings became easier and painkillers have been stopped. Interestingly, patient 2 gained weight (+6 kg within 3 months) and height (+4 cm during the same period). Particularly remarkable, the peripheric scalp is completely re-epidermized. Still the epidermal covering is not fully cornified, which fits with the absence of rescue of cornified specific markers, such as LOR and SPRRA1. This suggests that PRIMA-1^MET^ could enhance mainly early epidermal differentiation.

The localization of skin erosions in AEC patients could differ among patients, even with the same mutation on the SAM domain. Moreover, the erosion is always restricted to a well-defined area (see Fig. 4a), and skin biopsy in healthy area heals normally in AEC patients. There is no explanation for this puzzled fact. It is known that dermal fibroblasts are a heterogeneous cell population over the body, and they originate from different embryonic precursors. In addition, some skin areas contain more or less sweat and sebaceous glands, as well as particular ion (sodium, calcium) channels that could encounter for these differences. Finding the molecular basis for such restricted and variable insults among AEC patients would help to prevent such painful and disabiliting skin erosions. We showed that repurposing of PRIMA-1^MET^, a small compound identified as p53-reactivating drug to induce cell apoptosis of human cancer cells carrying a p53 mutation, could become an efficient treatment for local AEC erosions reported in patients with AEC syndrome. In addition, such treatment might improve wound healing. It might help the multidisciplinary teams to propose appropriate and early care that are limited by the lesions, i.e., clefting surgery, hearing aids. Chronic pain might be avoided. Finally, it improves quality of life and social networking in AEC patients. It remains to test whether PRIMA-1^MET^ must be applied permanently or temporary and to clarify the mechanism of action of PRIMA-1^MET^ on differentiated KCs.

## Materials and methods

### Cell culture and epidermal differentiation

Skin biopsies of 4 mm were taken from the back arm of patients 1 and 2 after family consent and authorization from the French Committed of Person protection (CPP). Primary KCs were extracted and amplified as described previously^15^. By raising calcium concentration to 1.5 mM at confluency, KCs underwent stratification/differentiation for 10 days. Cells were treated with 30 μM of PRIMA-1^MET^ dissolved in keratinocyte medium 2 days before induction of differentiation and during the whole period of treatment, with change of medium every 2 days.

### Formulation of PRIMA-1^MET^

Since i.v. injections of *PRIMA-1*^*MET*^*/APR-246* were well tolerated in previous clinical cancer trials (phase 2 and 3), the French agency for health and drugs (ANSM) gave us the authorization to treat by topical dermal application two AEC patients with severe skin erosions as a compassionate care, under the responsibility of the dermatologist and the pharmacist. Quality control of *PRIMA-1*^*MET*^*/APR-246* pure powder (R&D Systems, Bio-Techne, France) was assessed by appropriate assays. No impurities were found beyond the recommended thresholds of the ICH Q3A, Q3C, and Q3D guidelines. *PRIMA-1*^*MET*^*/APR-246* powder was dissolved in sterile purified water (Versylene^®^, Fresenius Kabi, France) to obtain a solution at 36 mg/mL. This solution was then incorporated and mixed in the ready-to-use Seraqua^®^ hydrophilic topical cream base (Fagron, France) to obtain *PRIMA-1MET/APR-246* concentration at 0.6 mg/g of cream. Stability of *PRIMA-1*^*MET*^*/APR-246* in this formulation was demonstrated for 3 weeks when stored at 2–8 °C.

### qRT-PCR analyses

Untreated and treated AEC and control KCs were harvested as a dry pellet. RNA was then extracted using RNEasy Mini kit (Qiagen), and cDNA was synthesized from 1 µg of RNA using iScript cDNA synthesis kit (Bio-Rad). Quantitative PCR was performed in triplicate using 2× SYBR Green PCR Master Mix (Absource Biotools). Expression of each gene was calculated using the 2^−ΔΔCt^ method. The results are presented as fold change normalized to B2M house-keeping gene and relative to control KCs. Specific primer sequences used are

KRT14: 5′-GGCCTGCTGAGATCAAAGACTAC-3′ (up) and 5′-CACTGTGGCTGTGAGAATCTTGTT-3′ (down)

KRT1: 5′-TCTCGGTTGGATTCGGAACTGAAG-3’ (up) and 5′-AGACAACTCTGCTTGGTAGAGTGC-3′ (down)

ZNF750: 5′-CAGGTACTGCTTCCTGAGCAC-3′ (up) and 5′-GAGAGCCTCCGTCATCTGG-3′ (down)

TGM1: 5′-CCCCCGCAATGAGATCTACA-3′ (up) and 5′-ATCCTCATGGTCCACGTACACA-3′ (down)

ILV: 5′-TCTGCCTCAGCCTTACTGTG-3′ (up) and 5′-CAGTGGAGTTGGCTGTTTCA-3′ (down)

B2M: 5′-CCACTGAAAAAGATGAGTATGCCT-3′ (up) and 5′-CCAATCCAAATGCGGCATCTTCA-3′ (down).

### Immunofluorescence staining

Cells were seeded on 0.1% gelatin-coated coverslips in a 24-well plate at 5400 cells per well. They were fixed after 10 days of differentiation with 4% paraformaldehyde for 20 min at room temperature, incubated for 10 min in glycine 1 mM to quench PFA and permeabilized with 0.5% Triton X-100 in DPBS^+/+^ (Gibco™, Life Technologies) for 7 min with 3 × 5 min of washing in DPBS^+/+^ between each step. After blocking in 5% BSA for 30 min, cells were incubated with primary antibodies overnight at 4 °C in a humidified chamber. Primary antibodies used were against CKRT1 (1/250, BioLegend), ZNF750 (HPA023012, 1/300, Sigma Aldrich), TGM1 (sc-25786, 1/50, Santacruz), and IVL (I9018, 1/100, Sigma). Cells were washed in DPBS^+/+^ and incubated with corresponding secondary antibodies (goat anti-rabbit AlexaFluor^®^ 488 or goat anti-rabbit AlexaFluor^®^ 594, Life Technologies) diluted at 1/3000 in blocking buffer for 1 h at room temperature protected from light. Coverslips were finally washed, mounted on microscope slides (DAPI fluoromount-G™, Electron Microscopy Sciences), and visualized under a Nikon Eclipse Ti epifluorescence microscope equipped with an OrcaFlash 4.0 LT camera (Hamamatsu). Picture analyses were conducted using NIS-Elements software.

### Protein aggregation tests

In total, 5 × 10^5^ AEC and control KCs were seeded in six-well plates. As they became 70% confluent, they were treated with 30 μM PRIMA-1^MET^, or the vehicle as control, replacing the culture medium with the compound every 48 h until the harvesting. At 100% of confluence, cells underwent differentiation by raising calcium concentration of the medium to 1.5 mM. After 10 days of differentiation, cells were lysed in native lysis buffer (25 mM Tris (pH 7.5), 150 mM NaCl, 2 mM MgCl_2_, 20 mM CHAPS, 1 mM DTT, and protease inhibitors) and incubated for 1 h on ice in the presence of benzonase (Merck Millipore). Protein extracts were loaded on 3–12% Novex Bis-Tris gradient gel for BN-PAGE (Life Technologies) in 20% glycerol and 5 mM Coomassie, and analyzed by western blotting using p63-specific antibody (anti-p63EPR5701, ab124762, Abcam). Relative quantification of protein band intensity was performed using Image Lab Software (Bio-Rad).
